# Biomechanics Assist Measurement, Modeling, Engineering Applications, and Clinical Decision Making in Medicine

**DOI:** 10.3390/bioengineering10010020

**Published:** 2022-12-22

**Authors:** Qingjia Chi, Pengchao Liu, Huaping Liang

**Affiliations:** 1 Department of Engineering Structure and Mechanics, School of Science, Wuhan University of Technology, Wuhan 430070, China; 2 State Key Laboratory of Trauma, Burns and Combined Injury, Department of Wound Infection and Drug, Daping Hospital, Army Medical University, Chongqing, 400042, China

Biomechanical studies of surgeries and medical devices are usually performed with human or animal models. Biomechanical models can quantitatively access information that is difficult to obtain experimentally, such as changes in strain rates, and have a wide range of applications [1]. Michele et al. [2] proposed a new surgical technique for treating primary bladder neck obstruction and maintaining paracolic ejaculation based on numerical simulations. The method improved urination, lower urinary tract symptoms, as well as sustained continual ejaculatory function and quality of life. Moreover, biomodelling and FEA enhanced an understanding of the mechanobiology of the heart. In turn, the differences in elastic modulus for physiological and pathological myocardium can be measured and analyzed to support mechanical conditioning and myocardial regenerative medicine in cardiac tissue engineering [3].

Finite elements serve as a relatively simple and effective method for studying biomechanics. Therefore, the computational biomodelling of structures such as the human heart and joints using finite element analysis (FEA) studies is arguably a critical method for studying the mechanical properties of human organs in various situations. Biomodelling analysis can assist in designing and manufacturing multicellular spheres, biocomposite scaffolds for bone tissue regeneration, and structural bone stimulations for muscle bionics [4,5,6]. The analysis helps us understand the properties of various parts of the human body and provides better treatment for patients.

The application of biomechanics has achieved specific achievements. Computational biomodelling and finite element analysis have been widely used in biomechanics. The heart is one of the more complex organs in human beings, and its mechanical properties are very complex, making treating various heart diseases difficult. The tricuspid valve (TV) consists of three leaflets that engage during systole to prevent deoxygenated blood from re-entering the right atrium. TV and atrioventricular heart valves (AHVs.03) are both dynamically remodeled tissues. Biomechanical properties are particularly important in the design of both biomaterials to promote cardiac tissue repair [3,7,8].

Authors have investigated the mechanical properties of the respiratory system through modelling, providing a basis to assist in clinical decision-making. The main determinant of airway mechanical properties is collagen, the most abundant extracellular matrix component of the airway. Abnormal airway collagen deposition is associated with the pathogenesis and progression of airway diseases, such as emphysema. A key feature is the destruction of the extracellular matrix (ECM) of the lung parenchyma, leading to dramatic changes in the mechanical properties of the lung. Respiratory modeling provides insight into the mechanical properties of the lungs and helps clinicians select different mechanical ventilation settings for different patients’ lung conditions in intensive care [9,10,11]. Thus, the technique can significantly improve the management of patients with respiratory diseases. Currently, the most developed models are only apply to fully sedated patients rather than patients with asynchronous breathing. Studies detected asynchronous breathing in a hospital’s intensive care unit for patients with MV using an extended time-varying elasticity model, which may guide clinicians in determining the optimal ventilation mode for stroke patients [12]. Follow-up studies still need biomechanics to improve models and treatments for respiratory diseases, especially emphysema.

Authors have studied the mechanical behavioral properties of the digestive system through a combination of computational modeling and experimentation, which has informed doctors in their surgical decisions. In recent years, rising living conditions have led to an increasing number of people developing symptoms of obesity. Although bariatric surgery (BS) is the most effective treatment for severe obesity, there are still drawbacks and complications as the intervention design is based mainly on the surgeon’s expertise and intraoperative decision-making. Ilaria et al. [13] developed a biomechanical model of the stomach considering the anisotropic viscoelasticity, nonlinear elastic response, and time-dependent behavior of the tissue. The model can provide information on gastric volume and stiffness to aid the surgeon in preoperative decision making. At the same time, diseases of the digestive system can also have an impact on obesity. Dimitrios et al. [14] performed multi-axial tests of the small intestinal wall using rat small intestinal wall tissue. The rat tissue’s nonlinear hyperelastic and anisotropic response was characterized by a fiber-reinforced model, reflecting the biomechanical properties of the small intestinal wall. To stimulate the growth and elongation of the small intestine, self-expanding springs that exploit biomechanical forces have been used. Hosseini et al. [15] developed a series of computational models based on experimental measurements of patient biometrics and mechanical properties of soft tissues to predict the response of individual tissues to spring-mediated detrusor heterogenesis. They can be used to safely deliver mechanical forces. In turn, many lower gastrointestinal disorders are associated with altered mechanical motion and deformation of the large intestine. However, the authors are still exploring the link between biomechanical models and mechanosensitive nerve endings to predict organ-level biomechanical regulation [16,17].

Measuring mechanical properties is difficult in conventional medicine and can be well addressed by biomodelling. Nguyen-Truong et al. [8] provided a multi-scale model that can better simulate the mechanical, microstructural, and morphological characteristics of the tricuspid leaflets. Nguyen-Truong et al. [18] measured mechanical properties of the left ventricular and septal side and ventricular wall, providing essential knowledge about septal wall biomechanics. The highly flexible and tough heart valve demonstrated complex mechanical characteristics, which are determined by the microstructure of the tissue components, particularly the collagen fibers. Navid et al. [19] used melt electro-writing (MEW) to create a functional scaffold with a highly controlled fibrous microstructure that mimics the load-dependent recruitment of collagen fibers. The biocompatible scaffold has strictly nonlinear and anisotropic mechanical properties required for HVTE.

Biomechanically manufactured materials, such as stents and implants, can assist in treating patients. The two main causes of separation within the root canal of endodontic instruments are cyclic fatigue and torsional loading. The biocompatible, superelastic, and shape memory properties of nickel–titanium alloys can promote the predictability and effectiveness of endodontic treatment and the success of root canal therapy [20]. At the same time, tissue engineering and regenerative medicine rely extensively on biomaterial scaffolds. Moreover, the study of scaffold surface properties is essential, significantly impacting cellular responses [21]. Post-implantation complications cannot be ignored in implantation surgery. A computational framework based on finite element analysis (FEA) to simulate patient-specific implantation as an adjunct to CT scanning can potentially predict post-implantation complications. It can provide relevant information for patient treatment [22].

Biomechanics also supports medicine by measuring human tissue’s mechanical parameters and refining its mechanical properties. The initial tension of the anterior cruciate ligament (ACL) in other muscles or tendon reconstructions is one of the critical factors affecting postoperative outcomes. However, it is rather difficult to measure tension after graft fixation. An appropriate quantitative intraoperative assessment is performed by pulling the soft tissue of the joint with an arthroscopic probe. Hananouchi et al. [23] also developed devices that can quantitatively measure tension in the reconstructed ACL after fixation and provide support for post-graft treatment.

Recent advances in medicine have greatly improved the management of the disease. However, the human body is a large and complex system. There are still various difficulties in the diagnosis and treatment of both cardiac and other diseases, such as respiratory diseases [3,10]. Consequently, biomechanics guides engineering applications to produce better models fitting the human body. Consequently, bioengineering has emerged and developed over a short period of time. Still, it has been subject to various technological advances from various disciplines, giving rise to various options available to produce complex geometrical structures to precisely manipulate and control cell behavior and to create complex kinetic models of living tissue [24].

With their short cycle time, safety, and low cost, biomechanics can be an excellent alternative to experiments and can be widely used in engineering. The menisci of the knee are complex fibrocartilaginous tissues that play an essential role in weight bearing, shock absorption, joint lubrication, and stability [25]. Ferroni et al. [25] assessed the interactions between different meniscal tissue components by numerical simulations. They revealed changes in the structural components of the tissue during maturation based on the mechanical response of the tissue developmental. Brasinika et al. [26] enhanced the mechanical properties of a bionic bone scaffold with a bone-like nanostructure and composition. Hamandi et al. [27] investigated the mechanics of tibial heel structures using the PHILOS electroplating system to provide a basis for their longevity and strength. Tse et al. [28] integrated an airbag system into a conventional helmet for a new bicycle helmet. They carried out a series of dynamic impact simulations of the helmet and found that it provided sufficient protection even when the airbag failed to deploy.

Biomechatronics (bionics) is an applied science that establishes an interdisciplinary link between biomechanics and engineering and is widely used today. The mechanical properties of innovative tissue-engineered bionic hydrogels based on hydrophilic polymers were investigated [29]. Investigators have developed a fecal mimic for studying defecation patterns in large animals and humans that can be used to help patients with defecation disorders and fecal incontinence symptoms. Simulated feces can possess the consistency and shape of normal feces, and various parameters have been recorded, including pressure, curvature, and shape changes [30]. Comunale et al. [31] compared three aortic models through a series of fluid–solid coupled simulations, reproducing patient-specific geometries with biological tissue or silicone walls based on in vivo data made of silicone. The replica with the same compliance as the real aorta can simulate the vessel’s overall behavior.

Biomechanics, which provides a good insight into the mechanical properties of various parts and tissues of living organisms, is an important part of medical science and provides the fundamental theoretical knowledge of mechanics for medical science. Bionics has tremendous advantages for specific patients, and bionic scaffolds that mimic the properties of natural bone tissue hold great promise for bone regeneration in tissue engineering applications. Adjustable bionic scaffold biomaterials offer more significant advantages for bone tissue engineering [26,29]. Analysis of the role of collagen in airway mechanics quantifies macro- and micro-scale approaches to airway mechanics and pathological changes associated with collagen deposition in airway disease in the quest to treat airway pathology and address airway defects [9]. In contrast, cellular and molecular biomechanics is a promising biomarker for early cancer diagnosis and prognosis [32]. It can inform the treatment of cancer cells through methods such as cell culture [33]. Meanwhile, advanced experimental and computational biomechanics has become an important component for understanding the physiological and pathological conditions of human biological tissues. Recent advances in medical imaging modalities, image segmentation, tissue characterization experiments, and predictive models significantly transform the therapeutic paradigm to facilitate patient-specific diagnosis and individualized surgical planning [34,35].

Finite elements and biomodelling enable a more comprehensive understanding of the mechanical properties of various tissue components and the appropriate use of medical devices and implants. Biomechanics helps to provide a precise model of complex tissues, improves the quantitative measurement of mechanical properties, offers some assistance to engineering applications, and aids in the clinical management of the patient. Based on biomechanics, it promises to produce more complex and realistic models of living tissues to aid accurate clinical decision-making.
